# Refractory hypertension complicated with Turner syndrome: A case report

**DOI:** 10.1515/biol-2022-0934

**Published:** 2024-08-06

**Authors:** Sisi Hu, Jichun Liu, Haixia Tang, Xiangrong Xie, Youquan Wei

**Affiliations:** Department of Cardiology, The First Affiliated Hospital of Wannan Medical College, Wuhu, 241000, Anhui, China

**Keywords:** hypertension, Turner syndrome, sex chromosome disorder

## Abstract

Hypertension is commonly classified into essential hypertension and secondary hypertension, although definitive classification can be challenging in some cases. Here, we discussed a patient who admitted for refractory hypertension, exhibiting various clinical manifestations including inadequate estrogen secretion, underdeveloped secondary sexual characteristics, primary amenorrhea, short stature, multiple moles, and somatic abnormalities. The patient was finally diagnosed with Turner syndrome (TS) based on clinical findings and chromosomal analysis. The genetic karyotype identified was 46,X,i(X)(q10).

## 1 Background

Turner syndrome (TS), also known as congenital ovarian dysgenesis, was first described by American endocrinologist Henry Turner in 1938. It is a chromosomal disorder characterized by complete or partial loss of one X chromosome in affected individuals. Its clinical manifestations include insufficient estrogen secretion, underdeveloped secondary sexual characteristics, primary amenorrhea, short stature, multiple moles, spinal deformities, and coarctation of the aorta [1]. Current data indicate an incidence of 1 in 2,000 live female births [2,3]. Individuals with TS face a threefold higher risk of cardiovascular diseases compared to the general population [4]. Hypertension emerges as a significant risk factor for acquired cardiovascular diseases, with females affected by TS prone to developing hypertension during childhood and adolescence. As reported by observational studies, the prevalence of hypertension among TS patients varied from 21 to 40%, increasing with age [5]. This report presented a rare case of refractory hypertension complicated by TS. Our study was aimed to explore the clinical characteristics, diagnostic challenges, and therapeutic strategies associated with this case, thereby enhancing understanding of this condition and providing a valuable reference for effective management for it.

## 2 Case presentation

On February 16, 2023, a 31-year-old female patient was admitted to the hospital due to a 1-day history of elevated blood pressure.

### 2.1 Medical history

The patient had visited the dental department a week before due to toothache resulting from dental caries. During this visit, her blood pressure was recorded as 220/120 mmHg, prompting the dentist to recommend consultation with the cardiology department. The patient reported transient dizziness and discomfort, with the first occurrence 3 months ago and a second occurrence 3 days ago. Although the patient did not consider these episodes significant, they were noteworthy. She denied any history of chronic illnesses. Despite being married for 5 years, she experienced irregular menstruation and remained nulliparous.


**Informed consent:** Informed consent has been obtained from all individuals included in this study.
**Ethical approval:** The research related to human use has been complied with all the relevant national regulations, institutional policies and in accordance with the tenets of the Helsinki Declaration, and has been approved by the authors’ institutional review board or equivalent committee.

### 2.2 Physical examination

Upon examination, her blood pressure in the right upper limb was observed to be 270/164 mmHg, and in the left upper limb, it was 262/158 mmHg. Her body mass index (BMI) was 25.33, with the weight of 54 kg and height of 146 cm, suggesting obesity according to standard BMI classifications. Coarse breath sounds were auscultated bilaterally, without the presence of crackles or wheezes. Her heart rate was 121 beats per minute, demonstrating a regular rhythm. No pathological murmurs were detected during auscultation of the cardiac valves. There was no edema observed in either lower limb.

### 2.3 Laboratory tests

**Figure 1:**
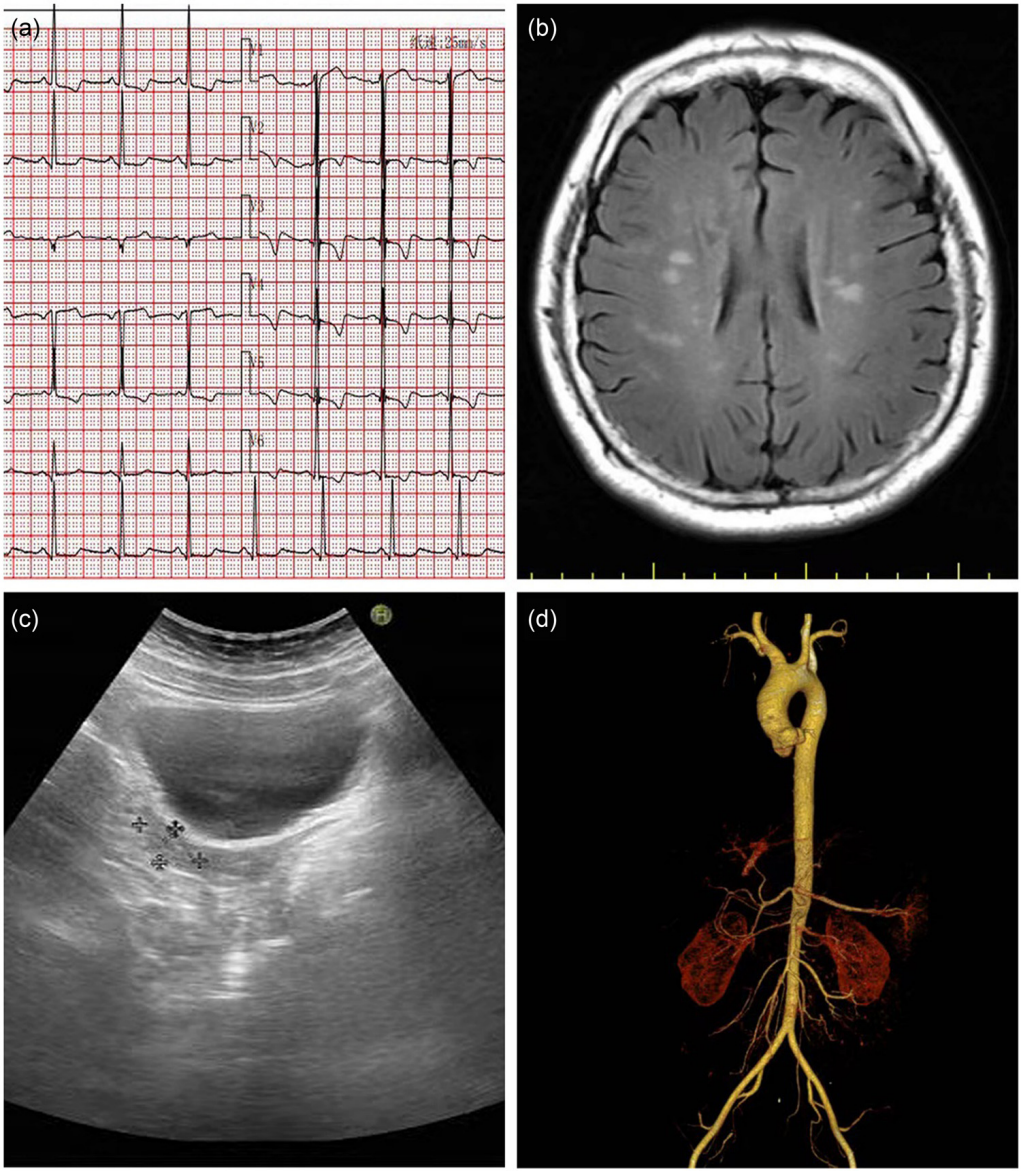
(a) Electrocardiogram showing sinus rhythm, left ventricular hypertrophy, and ST-T changes. (b) Acute cerebral infarction. (c) Gynecological color Doppler ultrasound suggesting uterine size of 23, 13, 28 mm and bilateral adnexal linear changes. (d) Chest and abdominal CTA showing no abnormalities.

The patient’s laboratory results indicated a potassium level of 2.71 mmol/L, creatinine of 145.7 µmol/L, urine protein of +++, anti-thyroglobulin antibody of 106.1 IU/mL, and thyroid peroxidase antibody of 131.4 IU/mL, suggesting hypokalemia, chronic renal insufficiency, and autoimmune thyroiditis. An electrocardiogram revealed sinus rhythm, left ventricular hypertrophy, and ST-T changes (Figure 1a). Cardiac ultrasound showed an ejection fraction of 58%, left atrium diameter of 42 mm, and left ventricle diameter of 43 mm, with normal valve morphology and closure. A head computed tomography (CT) indicated acute cerebral infarction (Figure 1b). Ambulatory blood pressure monitoring revealed a non-dipping pattern. The patient presented with hypertension and hypokalemia, and reported dental decay and toothache. Additionally, she experienced multiple episodes of vomiting following anesthesia rinsing before tooth extraction, suggesting that the hypokalemia may be secondary to vomiting. Oral potassium supplementation was promptly administered due to severe hypokalemia upon admission, and no 24 h urine potassium collection was performed. Further imaging studies, including adrenal and renal Doppler ultrasound, contrast-enhanced adrenal CT, and thoracoabdominal computed tomographic angiography (CTA), showed no significant abnormalities (Figure 1d).

### 2.4 Detailed findings

**Figure 2:**
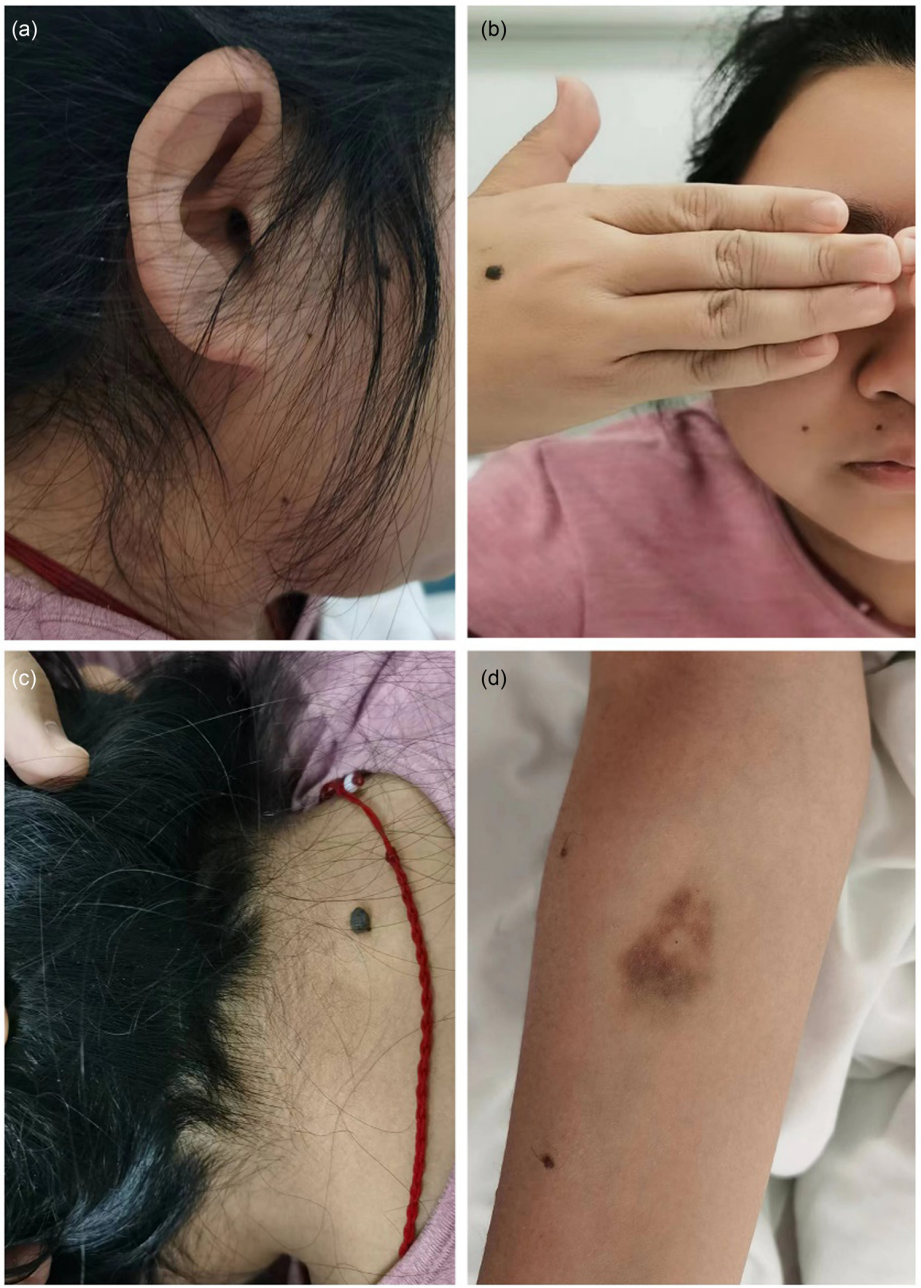
Multiple moles (a)–(d), webbed neck (c), and elbow pronation (d).

A comprehensive medical history and physical examination revealed the absence of secondary sexual characteristics, onset of menarche at 18 years old, irregular menstruation, 5 years of infertility, and previous gynecological examinations indicating a small uterus. Subsequent pelvic ultrasound showed a uterine size of 23 mm × 13 mm × 28 mm with bilateral adnexal changes (Figure 1c), suggesting possible premature ovarian failure. Further physical examination identified underdeveloped secondary sexual characteristics, short stature (146 cm), multiple moles, cubitus valgus, wide-set eyes, and ptosis (Figure 2). Hormonal analysis demonstrated decreased estradiol levels (14 pg/mL) and elevated follicle-stimulating hormone (FSH) (82.59 mIU/mL).

### 2.5 Discharge medications

The patient was discharged on the following medications: sacubitril valsartan sodium tablets 300 mg once daily, nifedipine sustained-release tablets 10 mg once daily, hydrochlorothiazide tablets 12.5 mg once daily, carvedilol 20 mg twice daily, enteric-coated aspirin tablets 0.1 g once daily, and atorvastatin calcium tablets 20 mg once nightly. During a follow-up visit 1 month later, an outpatient examination was conducted, which showed no presence of urinary protein detected and a creatinine level of 133 µmol/L.

## 3 Discussion

**Figure 3:**
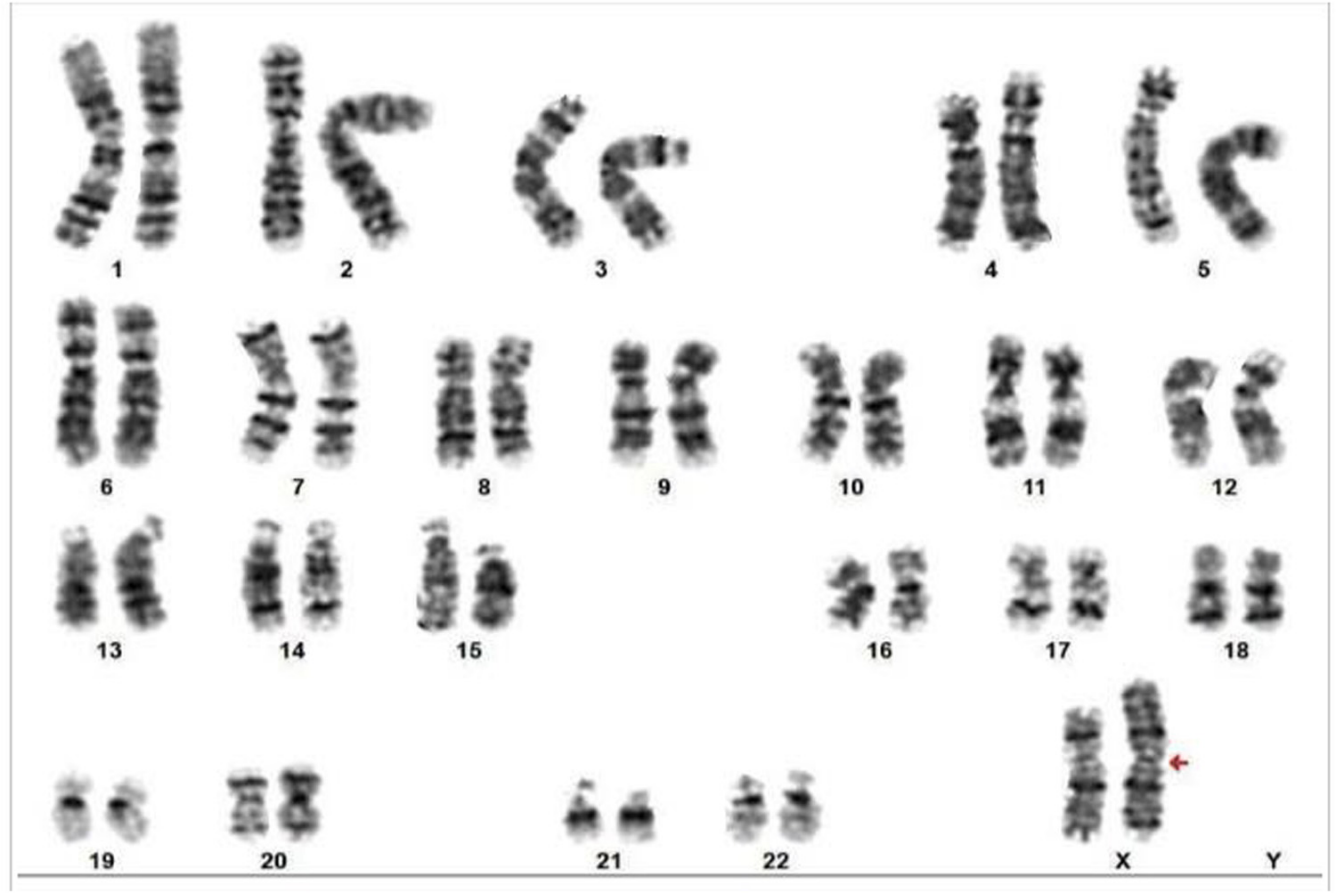
Karyotype: 46,X,i(X)(q10); the chromosome number: 46; the sex chromosome: X (missing one X chromosome compared to a normal female but with an additional structurally abnormal chromosome, i(X)).

The patient was a young female diagnosed with hypertension, initially measured at 220/120 mmHg. Given her age and lack of risk factors for essential hypertension, secondary causes were thoroughly investigated. Auxiliary examinations revealed multi-organ damage involving the heart, brain, and kidneys, suggesting potential secondary hypertension. Additionally, she presented with hypokalemia, prompting consideration of secondary causes such as primary hyperaldosteronism. However, her renin level was elevated at 138.30 µIU/mL, and aldosterone was also elevated at 58.10 ng/dL, indicating secondary hyperaldosteronism. Further imaging studies, including adrenal and renal Doppler ultrasound and contrast-enhanced adrenal CT, showed no significant abnormalities. Thoracoabdominal CTA ruled out the possibilities of coarctation of the aorta, renal artery stenosis, renin-secreting tumors, and primary hyperaldosteronism as potential causes of secondary hypertension (Figure 3).

Furthermore, the patient exhibited underdeveloped secondary sexual characteristics, menarche at 18 years with irregular menstruation, and a small uterus, prompting consideration of congenital adrenal hyperplasia (CAH), specifically 17α-hydroxylase deficiency, a chromosomal disorder. However, CAH typically presented with excessive androgens causing masculinization in females, including hirsutism, acne, irregular menstruation, and may involve underdeveloped or absent gonads, low cortisol levels, and elevated adrenocorticotropic hormone (ACTH) levels. This diagnosis was ruled out due to the patient’s normal cortisol and ACTH levels, as well as negative genetic testing for CAH (21-hydroxylase deficiency). Subsequent examinations revealed characteristic features such as short stature, multiple moles, elbow deformity, wide-set eyes, ptosis, and underdeveloped secondary sexual characteristics. Gynecological ultrasound showed a small uterus and premature ovarian failure, suggesting TS. Hormonal analysis showed decreased estradiol levels (14 pg/mL) and elevated FSH levels (82.59 mIU/mL), consistent with the presentation of TS. The diagnosis was confirmed through chromosomal testing, revealing a karyotype of 46X,i(X)(q10).

TS, also known as congenital ovarian dysplasia syndrome, is a chromosomal disorder characterized by distinctive features. Individuals with TS typically exhibit either complete or partial loss of one X chromosome. In the presented case, there was a mosaic/structural abnormality type with the loss of one X chromosome compared to the typical female karyotype, alongside an additional structurally abnormal i(X)(q10) chromosome. Despite clinical similarities to the 45,X0 karyotype (complete loss of one X chromosome), the patient’s symptoms did not align with typical presentations of this karyotype. Research indicates that individuals with TS, especially those with a 45,X0 karyotype, have a high incidence of congenital heart structural abnormalities, affecting up to 50% of cases [4,6]. However, no anomalies were observed in the patient’s echocardiogram or chest-abdomen CTA. Cardiac abnormalities associated with the 45,X0 karyotype often involve the left side of the heart, including issues with the mitral valve, aortic valve, aortic arch anomalies, progressive dilation of the ascending aorta, and aortic coarctation [5,7,8]. The patient experienced menarche at 18 years old, displaying irregular menstruation and exhibiting a small uterus. These clinical features strongly indicate the occurrence of premature ovarian failure, a condition that is highly prevalent in individuals with TS. In a study involving 522 TS patients aged over 12 years, only 14% of those with a 45,X0 karyotype experienced spontaneous thelarche, with higher rates observed in patients with mosaic karyotypes [9].

Extensive research has consistently shown that approximately 30–40% of individuals with TS exhibit congenital abnormalities in the urinary tract. Among them horseshoe kidneys and duplicated renal pelvis with duplicated ureters are the most commonly observed anomalies [10,11]. However, the patient under discussion did not present any congenital structural abnormalities in the urinary tract. Regarding hormonal findings, the patient showed elevated renin and aldosterone levels, consistent with studies reporting that about half of TS patients exhibit significantly increased renin activity, potentially linked to heightened sympathetic nervous system activity [12,13]. Additionally, the patient displayed a non-dipper pattern in blood pressure rhythm, suggesting a possible association with nocturnal sympathetic nervous system dysfunction and alterations in sympathetic-vagal nerve tone [14].

The pathogenesis of hypertension in TS remains unclear [15], and is likely to be multifactorial, involving shared mechanisms with primary hypertension such as autonomic nervous system imbalance, vascular sclerosis, increased myocardial stiffness, estrogen deficiency, renal abnormalities, obesity, and reduced physical endurance [3]. Current treatment strategies for TS patients rely on clinical experience. Initial antihypertensive recommendations often involve drugs that inhibit sympathetic nervous system activity and reduce the risk of aortic dissection, such as beta-blockers and angiotensin-converting enzyme inhibitors/angiotensin receptor blockers [3]. Given the pathophysiological similarities to essential hypertension, calcium channel blockers and diuretics may also be considered for hypertension management in TS [16]. Studies have suggested that estrogen replacement therapy can modulate the renin–angiotensin–aldosterone system and potentially lower blood pressure in patients with ovarian insufficiency [17]. However, there is conflicting evidence regarding the effects of estrogen replacement therapy in women with TS. Some clinical studies indicate that estrogen replacement therapy may worsen endothelial function, leading to arterial stiffness and an increase in central arterial blood pressure [18]. Therefore, the benefits of estrogen replacement therapy in TS patients remain debated.

## 4 Conclusion

The findings from this case study offered valuable insights capable of guiding the diagnosis and treatment of similar patients in future clinical practice. In the context of young female patients presenting with hypertension, it is crucial to conduct a comprehensive evaluation for potential secondary causes. Particularly, if an individual exhibits clinical manifestations such as underdeveloped secondary sexual characteristics, irregular menstruation, short stature, multiple moles, cubitus valgus, wide-set eyes, and ptosis, TS should be considered. Timely genetic testing is essential to facilitate early identification and intervention, which can ultimately improve long-term prognostic outcomes for patients with TS.
